# Determinants of influenza and COVID-19 vaccine intent or uptake in Lebanon: a scoping review of the literature

**DOI:** 10.1186/s12879-023-08478-4

**Published:** 2023-08-06

**Authors:** Mahmoud Salam, Gladys Honein-AbouHaidar

**Affiliations:** Rafic Hariri School of Nursing, American University of Beirut, P.O. Box 11-0236, Beirut, 1107 2020 Lebanon

**Keywords:** Influenza, COVID-19, Vaccine, Hesitancy, Concept, Lebanon, Review

## Abstract

**Background:**

Vaccination is essential to protect from influenza and recently from COVID-19, yet uptake in Lebanon is suboptimal. Several factors determine uptake including knowledge, attitude and policies. We conducted a scoping review of the literature to explore the determinants of influenza and COVID-19 vaccine intent or uptake in Lebanon.

**Methods:**

Following the Preferred Reporting Items for Systematic Reviews and Meta-Analyses extension for Scoping Reviews (PRISMA-ScR) guidelines, thirty one peer reviewed studies indexed in six databases Pub Med, EMBASE, Scopus, CINAHL, Medline, and the Cochrane Library were screened. Two students, a senior Librarian and an Associate Professor in nursing searched for eligible studies. The library search strategy followed a combination of three broad concepts (viral influenzas; vaccines; Lebanon). The search timeframe was up till December 31, 2022. Determinants of influenza and COVID-19 vaccine intent or uptake were categorized following the constructs of the Theory of Planned Behavior.

**Results:**

Nine studies investigated influenza vaccine intent or uptake among the public community, parents and healthcare workers. Twenty one studies investigated COVID-19 vaccine intent or uptake among the public community, older refugees, university students, patients with cancer, dentists, and social media users. One study investigated both types of vaccines. A conceptual model of the determinants of vaccine intent and uptake within the Lebanese context was developed. Various determinants included environmental factors, norms, knowledge, perceptions, attitudes, past experiences, behavioral control and hesitancy.

**Conclusions:**

Research on vaccine intent and uptake in Lebanon is still in its infancy, while that of COVID-19 is on the rise. Multifaceted reasons behind the low vaccination rates were observed yet few attempts were made to target vulnerable groups. Further research studies are needed to target vulnerable groups.

## Background

The seriousness of the impact of both influenza and Corona Virus Infectious Disease (COVID-19) is notable. A survey from eight Lebanese hospitals showed that between 2018 and 2020, 30–40% of the 1,238 severe acute respiratory infections were due to viral influenzas [1]. During February of 2020, the weekly mortality rate due to influenza across Lebanon was between 20 and 50 deaths [1]. In terms of COVID-19, there have been more than 1.02 million confirmed cases of COVID-19 and 9,890 deaths in Lebanon as of February 2022 [2]. Unfortunately, determinants of influenza and COVID-19 vaccine uptake were not thoroughly explored and modeled within the Lebanese context. Exploring these determinants is of particular importance to the global community, the Lebanese Ministry of Public Health (MoPH), health care administrators and individuals as well. At the global level, the World Health Organization (WHO’s) position paper called for a number of recommendations including studies to understand the drivers of influenza and COVID-19 vaccine uptake in low and middle income countries such as Lebanon, a country that doesn’t meet the WHO recommendations to control for influenza transmission and decrease disease burden [3, 4]. At a national level, understanding the drivers and barriers of influenza and COVID-19 vaccine uptake fulfils an essential primary health care objective. It aids in designing/testing effective interventions to overcome poor influenza and COVID-19 vaccine uptake.

Vaccine is an essential measure to prevent the spread of viral influenzas [5, 6]. Yet, influenza and COVID-19 vaccine uptake rates in Lebanon, a Middle Eastern and North African (MENA) country are consistently suboptimal. They are estimated at less than 20 doses/1,000 compared to 275 doses/1,000 individuals in American countries [7]; thus not meeting the WHO recommendations [4]. Similar to influenza vaccines, COVID-19 vaccine uptake rates are also suboptimal. By June 19, 2023, the Lebanese MoPH stated that the cumulative rate of COVID-19 vaccine uptake in Lebanon was 2.75 million (single-dose recipients), 2.41 million (double-dose recipients), and 0.67 million (triple-dose recipients) [8]. However the percentage of COVID-19 vaccine coverage within the adult Lebanese population (≥ 12 years old) remains low (44.4% and 27.6% for double and triple dose recipients respectively) [8]. The Lebanese population seems to be lagging behind in terms of both influenza and COVID-19 vaccine uptake rates. For vaccines to have a desirable impact on the short-term, vaccine coverage should be at least 70% to avoid a less needed surge of pandemic in a country witnessing multiple crises and a limited health-care capacity [9].

Determinants of influenza and COVID-19 vaccine uptake in the MENA region were reported by many researchers [10, 11]. For instance, in terms of social influences and recommendations, a physician’s advice to get vaccinated against influenza and COVID-19 were associated with higher vaccine uptake rates in Iran [12–14]. In terms of risk perceptions, the perceived susceptibility / severity of influenza and COVID-19 were commonly reported in many MENA countries [15–17]. In some countries, mandates played a role in influencing people’s decision to get vaccinated against influenza and COVID-19. Other factors included socio-demographics, health-related, context-related, attitudes, hesitancy and others that varied between populations. Populations in the MENA region consist of diverse yet unique ethnic, linguistic, sectarian, familial, tribal, religious, socioeconomic, and national identities [18]. However, the socio-cultural fabric in Lebanon differs from its neighboring countries [19]. In Lebanon, the predominant culture is fundamentally conservative that exhibits a great deal of respect for traditions and customs, despite the influence of Western cultures on some life aspects [19]. This indicates that there is a need to explore the determinants of influenza and COVID-19 vaccine uptake within the Lebanese context.

The Strategic Advisory Committee on Immunization Practices (SAGE) - the principal advisor for WHO on the overall global vaccine policies and strategies - routinely revises the various determinants of vaccine uptake reported in literature [20–29]. Therefore the body of literature in Lebanon needed an evaluation on this matter. In addition, SAGE recommends more research on vaccine hesitancy in low to middle income countries and in under researched populations, to better understand its context-specific determinants [29]. Last but not least, influenza and COVID-19 vaccine uptake were not conceptualized within the Lebanese context.

Interestingly, the COVID-19 crisis influenced influenza vaccination negatively in some instances and positively in others. Prior the pandemic, the rate of influenza vaccine uptake among the general Lebanese public was 27.6%. In terms of COVID-19 vaccine intent, almost 59% of the Lebanese public community were willing to get vaccinated which showed that some Lebanese had a negative attitude towards the importance of COVID-19 vaccines [30]. The general public community in Lebanon might have missed on taking the quadrivalent seasonal influenza vaccines during the COVID-19 pandemic, while it motivated health care workers to get the influenza vaccine (flu vaccination uptake has risen from 32.1% in 2019–2020 to 80.2% in 2020–2021 [31]. There was a 12% increase in influenza vaccine uptake rate during the COVID-19 pandemic among Lebanese dentists [32]. This contradiction can be attributed to many factors. At the policy level, the Lebanese MoPH memorandum No. 149 (issued on October 6th, 2020) recommended influenza vaccines mainly to health care workers. In Lebanon, influenza vaccine itself was not included in the national immunization programs before or during the pandemic, neither mandated nor funded by the Lebanese MoPH. Moreover, the national data on annual influenza vaccine coverage in Lebanon was not clearly mapped and reported. At the primary health care level, more than half of the surveyed health care workers in one study did not properly identify candidate patients for influenza vaccination, and less than half of them did not promote for the importance of getting the influenza vaccine [31]. Insufficient amount of influenza vaccines was also a significant barrier reported by health care workers in the same study. The health care workers’ decision to get vaccinated against influenza was directly influenced by their fear of co-infection (influenza and COVID-19) and the recommendations of CDC [31].

Between January 2020 and December 2022, thirteen review studies addressed influenza vaccine related topics; whereas 24 review studies addressed COVID-19 vaccine related topics. It was evident to us there was no scoping review studies of the Lebanese literature to identify and model vaccine determinants. Between 2006 and 2016, one review study focused on influenza vaccine policies, use, recommendations and coverage within the MENA region [33]. This review only pointed to two studies from Lebanon. Therefore a scoping review study was needed to examine the extent and direction of the literature on influenza and COVID-19 vaccine status in Lebanon. Pinpointing the determinants of influenza and COVID-19 vaccine uptake in Lebanon is of particular interest to the Lebanese policymakers and researchers, and at a global level to international experts at SAGE who might be interested in the vaccination status in this country. Last but not least, a scoping review study is capable of pinpointing knowledge gaps related to influenza / COVID-19 vaccine status in Lebanon.

## Methods

This scoping review study followed the guidelines of the Preferred Reporting Items for Systematic Reviews and Meta-Analyses extension for Scoping Reviews (PRISMA-ScR) [34]. The stages of this study included: identifying the research question, identifying the relevant studies, selecting the studies, extracting the data, collating, summarizing, and synthesizing the results.

### Identifying the research question

The research questions included:

- What were the studies that investigated influenza and COVID-19 vaccine intent or uptake in Lebanon?
- What were the characteristics and outcomes of these studies?
- What were the determinants of influenza/COVID-19 vaccine intent or uptake in Lebanon?

### Identifying the relevant studies

Peer reviewed journals indexed in Pub Med, EMBASE, Scopus, CINAHL, Medline, and the Cochrane Library were screened. Studies published in subscription journals (restricted or paid access journals) were retrieved from the Saab Medical Library at the American University of Beirut. Two students, a senior Librarian and an Associate Professor in nursing searched for eligible studies. The students successfully completed an advance course on systematic reviews and meta-analyses. The library search strategy followed a combination of three broad concepts (viral influenzas; vaccines; Lebanon) (Table 1). Under each concept, specific medical subject headings (MeSH) terms were combined and searched in each data base using the common Boolean operators. The timeframe for the reviewed published studies was up to December 31, 2022. The date of data collection for each study was also inspected. The geographical space of the published studies was limited to Lebanon.

**Table 1:** Search terms

	MeSH terms	Boolean operators	MeSH terms
**Viral Influenza**	(MM “Corona virus Infections+”) OR (MM “COVID-19”) OR (MM “Coronavirus+”) OR (MM “SARS-CoV-2”) OR (MM “Caliciviridae Infections”) OR (MM “SARS Virus”) OR (MM “Middle East Respiratory Syndrome”) OR (MM “Middle East Respiratory Syndrome Coronavirus”) OR (MM “Coronaviridae+”) OR “Betacoronavirus” OR “Beta-coronavirus” OR (MM “Nidovirales+”) OR (MM “COVID-19 Pandemic”) OR “nCoV” OR “CoV” OR “Sars-cov” OR “sars” OR “Mers-related cov” OR “Mers-associated cov” OR “Mers-associated coronavirus” OR “Mers-related coronavirus” OR “Mers-Cov” OR “mers”	**(OR)**	(MM “Influenz*”) OR (MM “Influenz* A H5N1”) OR (MM “Influenz* A Vir*”) OR (MM “Influenz* A Vir*, H1N1 Subtyp*”) OR (MM “Influenz* A Vir*, H3N2 Subtyp*”) OR (MM “Influenz* A Vir*, H5N1 Subtyp*”) OR (MM “Influenz* B Vir*”) OR (MM “Influenzavirus C”) OR (MM “Influenz* B Vir*”) OR (MM “Orthomyxovirid*”) OR (MM “Severe Acute Respiratory Syndrome”) OR (MM “Respiratory Distress Syndrome, Acute”) OR (MM “Respiratory Distress Syndrome”)
		**(AND)**	
**Vaccine**	(MM “Immuniz*”) OR (MM “Immuniz* Schedule”) OR (MM “Immuniz*, Secondary”) OR (MM “Immuniz* Program*”) OR (MM “Immuniz*-Vaccination Administration (Iowa NIC)”) OR (MM “Immuniz* Behav* (Iowa NOC)”) OR (MM “Vaccin* Cover*”) OR (MM “Immunity, Herd”) OR “vaccin* refus*” OR (MM “Anti-Vaccin* Movement”)	**(OR)**	(MM “Influenz* Vaccin*”) OR (MM “COVID#19 Vaccin*”)
		**(AND)**	
**Lebanon**	“leban*” or “liban*” or “lubnan*” or “lobnan*”		

**MeSH: Medical subject headings**

### Selecting the studies

Studies retrieved from the databases were compiled into a reference software manager (Endnote x7). Duplicate studies were rapidly identified by Endnote, confirmed by the research team and then removed. Next, a first round preliminary review of titles and abstracts for the selected studies was performed based on the inclusion and exclusion criteria stated in Table 2. After excluding ineligible studies, the remaining studies were subject to a second round of thorough review (full version), to confirm that the selected studies met the eligibility criteria. The full texts of the remaining studies were retrieved using the Uniform Resource Locators (URLs).

**Table 2:** Inclusion and exclusion criteria

**Inclusion criteria**	
* Studies conducted in Lebanon * Studies that investigated vaccine intent or uptake * Studies that investigated seasonal influenza or COVID-19 vaccines * Peer-reviewed journals	
**Exclusion criteria**	
* Virology and sero-prevalence studies * Disease burden studies * Studies on vaccine development * Non-human research studies * Studies that did not report primary data (case studies; editorials, commentaries, brief reports, conference proceedings, systematic reviews and meta-analysis)	

### Extracting the data

The two students independently extracted data. Data extraction followed a structured tool developed by the research team and tailored to the study needs based on the work of Schmid et al. 2017. Schmid et al. conducted a large scale systematic review of 470 studies on influenza vaccine intent and uptake after screening 13,575 globally published studies from 13 databases [29]. The extracted data were compared and in case any dispute was encountered, it was resolved by a third investigator. The tool captured the study characteristics, i.e. the journal’s name/impact factor, the year of publication, the date of data collection, the setting, and the study design. The characteristics of the populations targeted by these studies were collected (such as age, disease, occupational groups or the general public). Influenza and COVID-19 vaccine intent and uptake were described. Studies that investigated vaccine intent were included since a number of studies on COVID-19 vaccines were conducted prior the introduction of these vaccines to the Lebanese market (2019–2020). Synonymous terms for vaccine uptake included vaccine rate, behavior, coverage, or compliance. Synonymous terms for vaccine intent included vaccine willingness, readiness, and registration. In case a study did not report vaccine intent or uptake, its main study outcomes were reported.

### Synthesizing the results

Determinants of both seasonal influenza and COVID-19 vaccine intent or uptake (at the bivariate and multivariate levels of analyses) were categorized under the constructs of the Theory of Planned Behavior (TPB) [29, 35]. The TPB is an *etic* approach theory that has been used to predict and explain a wide range of behaviors, including vaccination [36]. The reliability, validity, and model fitness of this theory have been reported to be significant in replete number of studies [29, 37–40]. The constructs of TPB include environmental factors (access to vaccines, quality of health care systems, policies, and other contextual factors related to influenza vaccination), social norms, socio-demographics and health related factors, knowledge, source of information, past experiences with influenza/COVID-19 and their vaccines, risk perceptions and attitudes, and the degree of behavioral control [41]. Any determinant extracted from the reviewed studies that did not align with these constructs was grouped in additional constructs, such as the constructs of vaccine hesitancy. Vaccine hesitancy has been conceptualized by the SAGE group into the ‘5 C’ vaccine hesitancy model with determinants related to confidence, complacency, constraints, calculation and collective responsibility [42, 43]. Determinants were also stratified by the vaccine type (seasonal vs. COVID-19 vaccines).

### Controlling of potential biases

Reviews are prone to potential biases, so every effort was made to control them. Study investigators revised all eligible studies and presented all possible determinants, thus eliminating any chance of committing evidence selection bias. Critical appraisal of studies passed through two sequential levels. Study investigators refrained from revising studies in which they were part of or in case they were in a direct relationship (social or professional) with their authors. This was disclosed among all study investigators a priori. Data were revisited and validated by the research team via random checks. The quality of the 20 screened journals was examined and its impact factors ranged between 0.2 and 4.96 as per the 2023 master journal list of the Web of Science and journal websites.

## Results

**Fig. 1:**
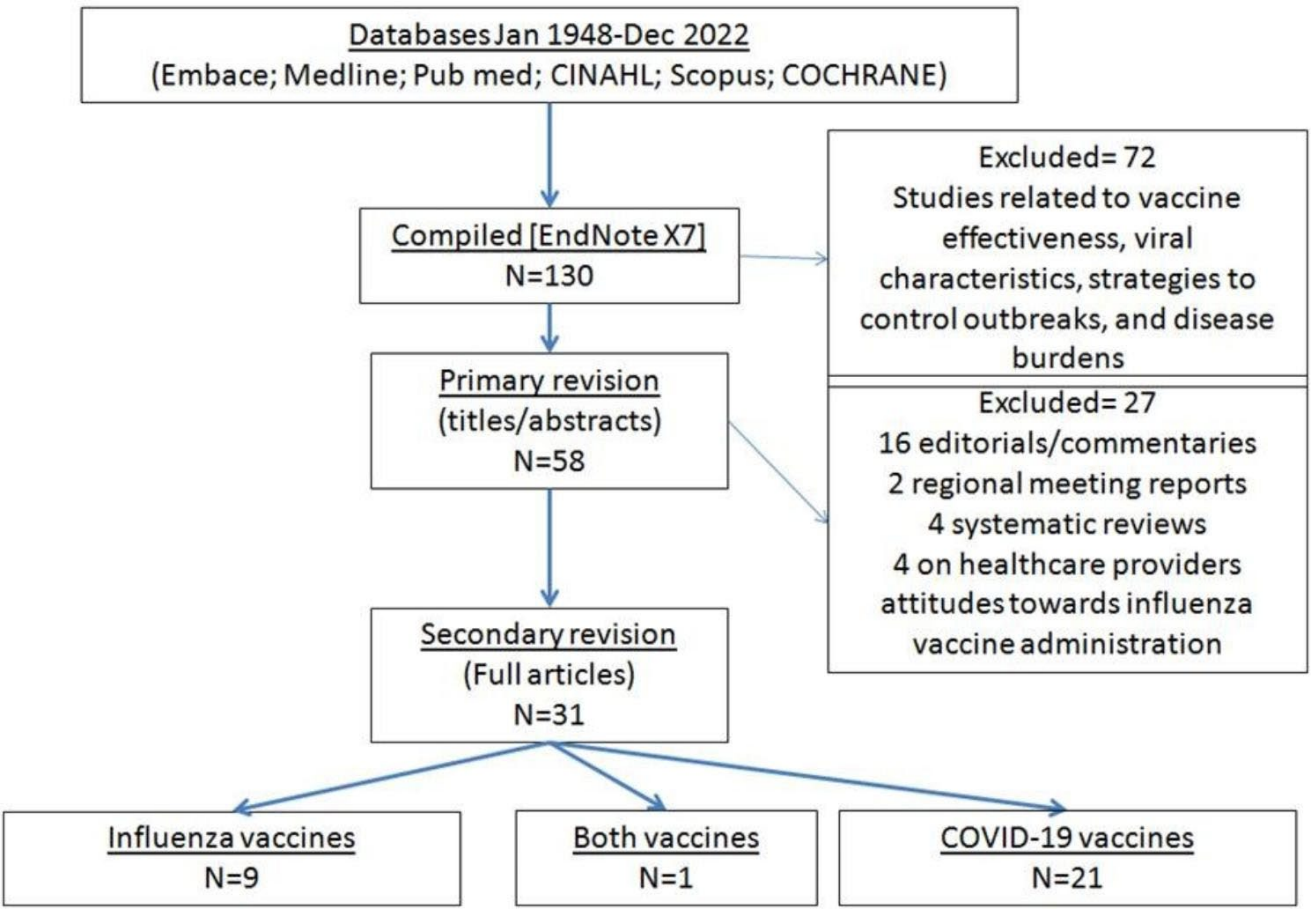
PRISMA flow chart

Up till the end of December 2022, 31 eligible studies were included in this review study (Table 3). As shown in the PRISMA flow chart, 99 studies were excluded (Fig. 1). Studies were excluded if they investigated vaccine effectiveness, viral characteristics, disease control strategies, and disease burden. Editorials, systematic reviews and meeting reports were also excluded.

**Table 3:** List of eligible studies included in the scoping review study

Study	Data collection date	Setting	Study measure	Target population	Sample size	Method of data collection
Seasonal influenza vaccines (n = 9 studies)						
(El Khoury & Salameh, 2015) [44]	2015	Pharmacies	Based on previous studies	General public adults including elderly adults and disease-specific groups	640	Face to face
Objective: Assess the rate of seasonal influenza vaccination among the Lebanese population; examine the knowledge and attitudes towards the influenza vaccine.						
(Taleb et al., 2018) [45]	2014	Conference	Based on previous studies	HCWs	227	Self-administered
Objective: Assess the health behavior including vaccine uptake among primary care physicians						
(Kmeid et al., 2019) [46]	2018	School	Based on previous studies	Children	571	Face to face
Objective: Evaluate vaccine compliance and the factors influencing the vaccination rate among Lebanese residents and Syrian refugees in infants and children up to 15 y of age						
(Tassi, 2020) [47]	2019	In Hospital	Based on previous studies	Elderly adults	125	Face to face
Objective: Assess the impact of educational program implementation on recognizing of influenza and adhering to vaccination						
(Moussa, 2020) [48]	2019	School	Based on previous studies	School students	370	Face to face
Objective: Test the effectiveness of a health education intervention in improving the knowledge, attitudes, and practices (KAP) toward influenza and its vaccine among secondary schools’ students in South Lebanon.						
(Choucair et al., 2021) [49]	2017	University	Based on previous studies	General public adults including HCWs	247	Self-administered
Objective: Determine the knowledge of, perception, attitudes, and behaviors toward influenza virus and immunization, and the determinants of vaccination among students, patients, and Healthcare Workers (HCWs) at the American University of Beirut and its affiliated Medical Center						
(Alame et al., 2021) [50]	2019	In Hospital	Based on previous studies	HCWs	429	Face to face
Objective: Assess factors associated with vaccine uptake and practices among HCWs in Lebanon						
(Zakhour et al., 2021) [51]	2018	School	Based on previous studies	School students	306	Face to face
Objective: Assess the rate of vaccination refusal and potential associated factors among Lebanese parents of School-aged children, in general and with a focus on influenza vaccine.						
(Dalal Youssef, Berry, et al., 2022) [31]	2020 Oct	Social Media Platforms	Based on previous studies	HCWs	560	Web based online
Objective: Evaluate the flu vaccination coverage rates among Lebanese HCWs and to assess their knowledge, attitudes, practices, perceived barriers, and benefits toward the flu vaccine during the COVID-19 pandemic, and to identify the factors associated with flu vaccine uptake						
COVID-19 vaccines (n = 21 studies + 1 study on both types of vaccines)						
(Abu-Farha et al., 2021) [52]*	2020 Dec	Social Media Platforms	Based on previous studies	General public adults including HCWs	2925	Self-administered
Objective: Assess the willingness of Middle Eastern Arab publics to receive COVID-19 vaccines and investigated the factors behind any reluctance to receive them						
(Kaadan, Abdulkarim, Chaar, Zayegh, & Keblawi, 2021) [53]*	2021 Jan	Community	Based on previous studies	General public adults including disease-specific groups	107	Web based online
Objective: Explore vaccine acceptance among Arab populations, and compare it with the global numbers.						
(C. Kasrine Al Halabi et al., 2021) [30]	2020 Dec	Community	VHS	General public adults including disease-specific groups	579	Self-administered
Objective: Assess the intent to receive the COVID-19 vaccine among Lebanese adults and the factors associated with vaccine refusal.						
(Sakr et al., 2021) [54]	2020 May	Community	Based on previous studies	General public adults including students; occupational groups	1861	Web based online
Objective: Determine the knowledge, attitude and practices (KAP) towards COVID-19 in Lebanon						
(M. Sallam et al., 2021) [55]*	2020 Dec	Community	VCBS	General public adults including disease-specific groups	9	Web based online
Objective: Assess the attitudes towards the prospective COVID-19 vaccines among the general public in Jordan, Kuwait and other Arab countries						
(Abd ElHafeez et al., 2021) [56] *	2020 Dec	Social Media Platforms	Vaccine Hesitancy Scale	General public adults including HCWs	24	Web based online
Objective: Translate, culturally adapt, and validate the 5 C scale into the Arabic language.						
(Aoun, Aon, Alshammari, & Moussa, 2021 [57] *	2020 Dec	Social Media Platforms	Based on previous studies	HCW	864	Web based online
Objective: Explore health care workers’ attitudes towards the COVID-19 vaccine and find the reasons lying behind vaccine hesitancy among participants.						
(Hamdan et al., 2021) [58]	2021 May	Social Media Platforms	Based on previous studies	University Mixed Students	3805	Web based online
Objective: Identify factors predicting behavioral intentions of students enrolled at the American University of Beirut to obtain a COVID-19 vaccine.						
(Nasr et al., 2021) [32]	2021 Feb	Social Media Platforms	Based on previous studies	Dentists	529	Web based online
Objective: Assess COVID-19 vaccination acceptance and its determinants among Lebanese practicing dentists						
(E. A. Qunaibi et al., 2021) [59]*	2021 Jan	Social Media Platforms	Based on previous studies	General public adults including disease-specific groups	56	Web based online
Objective: Measures vaccine hesitancy among Arab-speaking subjects.						
(E. Qunaibi et al., 2021) [60]*	2021 Jan	Social Media Platforms	Based on previous studies	HCW	59	Web based online
Objective: Assess the rates of COVID-19 vaccine hesitancy in Arabic-speaking HCWs residing in and outside Arab countries, and their perceived barriers towards vaccination.						
(Salibi et al., 2021) [61]	2021 Jan	Community	Based on previous studies	Older Refugees	1037	Telephone
Objective: Assess COVID-19 vaccine intentions among a sample of older Syrian refugee beneficiaries of a humanitarian organization in Lebanon, and explore factors associated with vaccine refusal						
(Elissar Moujaess et al., 2021) [62]	2021 Feb	Hospital	Based on previous studies	Patients With Cancer	111	Face to face
Objective: Assess the acceptance of the corona virus disease 2019 (COVID-19) vaccine among patients with cancer						
(Zeitoun et al., 2022) [63]	2021 Feb	Primary Health Care	VHS	Refugees	4174	Face to face
Objective: Evaluated variations in attitudes toward COVID-19 vaccines and factors associated with vaccine acceptance among refugees and Lebanese nationals						
(Biswas, Ali, Ali, & Shah, 2022) [64]*	2021 May	Social Media Platforms	Based on previous studies	General public adults including HCWs	217	Web based online
Objective: Identify if the social media usage factors can predict Arab people’s attitudes and behavior toward the COVID-19 vaccines						
(Ghaddar et al., 2022) [65]	2020 July	Medical Records	Based on previous studies	General public adults	1052	Telephone
Objective: Describe the trust in social media platforms and the exposure to fake news about COVID-19 in Lebanon and to explore their association with vaccination intent.						
(Ghazy et al., 2022) [66]*	2022 March	Social Media Platforms	HBM scale	General Public adults including disease-specific group, students and HCWs	2327	Web based online
Objective: Assess the acceptance of COVID-19 vaccine booster doses in low, middle, and high-income countries of the East Mediterranean Region (EMR) and its determinants using the health belief model (HBM).						
(P. Hanna, A. Issa, Z. Noujeim, M. Hleyhel, & N. Saleh, 2022) [67]	2021 Feb	Social Media Platforms	Based on previous studies	General public adults including pregnant women and disease-specific group	1209	Web based online
Objective: Asses COVID-19 vaccine acceptance and its related determinants in the Lebanese population						
(Jabbour et al., 2022) [68]	2021 June	Social Media Platforms	GHQ-12 and VAX scale	University Mixed Students	440	Web based online
Objective: Examine Lebanese University students’ perceptions of social media influence during the COVID-19 pandemic, as well as to measure the impact of misinformation on respondents’ mental health and vaccination decisions.						
(Karam et al., 2022) [69]	2020 June	Social Media Platforms	Based on previous studies	General public adults including disease-specific groups	352	Web based online
Objective: Investigate the willingness to pay (WTP) for a hypothetical vaccine and its associated determinants among the Lebanese general population during one of the peak episodes during the corona virus disease 2019 (COVID-19) pandemic in Lebanon.						
(Khatatbeh et al., 2022) [70]*	2021 Nov	Social Media Platforms	HBM	Parents / Children	424	Web based online
Objective: Assess childrens’ rate of COVID-19 Vaccination as reported by parents, to explore parents ‘attitudes towards children’s COVID-19 vaccination, and to examine the factors associated with parents’ hesitancy towards children’s vaccination in several countries in the Eastern Mediterranean Region (EMR).						
(Dalal Youssef, Berry, et al., 2022) [31]	2020 Dec	Social Media Platforms	HBM	HCWs	1800	Web based online
Objective: Assess the acceptance rate of the COVID-19 vaccine among HCWs and to identify its determinants.						

Health Belief Model (HBM); Health care workers (HCW); Vaccine Conspiracy Beliefs Scale (VCBS); Vaccine Hesitancy Scale(VHS); General health questionnaire (GHQ-12); Vaccine Attitude Scale (VAX); Multi-country study, Lebanon was a sub setting(*)

### Characteristics of the included studies

The authors’ names, the publication year, date of data collection and targeted populations are listed in (Table 3). Four studies were published in the journals “BMC Public Health” and “PLOS One” each. Three studies were published in the journal “Vaccines(Basel)”. The list of journals in which the studies were published is presented in Table 4. In terms of the date of publication, the majority of the eligible studies (n = 29) were published during the COVID-19 pandemic (2020–2022). More importantly, data from 23 studies were collected during the COVID-19 pandemic. Nine studies investigated influenza vaccine uptake, 21 studies investigated COVID-19 vaccine uptake, and one study investigated both types of vaccines. Out of the 31 studies, 9 studies were conducted in multiple MENA countries one of which included Lebanon. One study investigated COVID-19 vaccine booster uptake. The ultimate majority of the reviewed studies adopted a cross-sectional design. Most of the studies targeted the general public population (n = 13), among whom subgroups in some studies were analyzed (age or disease specific groups). Other studies targeted healthcare workers (n = 8) including physicians, dentists, pharmacists, nurses, and others. Few studies targeted children (n = 4), refugees (n = 2), patients with cancer (n = 1), elderly adults (n = 1), university students (n = 1). Few studies were guided by a theoretical model, such as the Health Belief Model (HBM), Integrated Behavioral Model (IBM), Precaution Adoption Process model (PAP) and the Extended Parallel Processing Model (EPPM) [31, 56, 58, 63, 66].

**Table 4:** Screened journals

Journal name	Number of studies
BMC Public Health (IF: 4.14); PLOS ONE (IF: 3.75)	4 each
Vaccines (Basel) (IF: 4.96)	3
Int. J. Environ. Res. Public Health (IF: 4.61); Human Vaccines & Immunotherapeutics (IF: 4.53); BAU journal	2 each
BJGP open; BMC Oral Health; eLife; Epidemiology and Infection; Future Medicine; Future Oncology; Glob Health Res Policy; Irish Journal of Medical Science; Journal of Epidemiol Glob Health; Journal of Pharmaceutical Policy and Practice; Oxford University Press; Preventive Medicine Reports; Saudi Pharm J; Sciencedirect; The Open Public Health Journal	1 each

Impact factor (IF)

### Study outcomes

The revised studies either reported vaccine uptake rate, vaccine registration, vaccine intent rate, vaccine acceptance or hesitancy, vaccine refusal or readiness to pay for a vaccine. As shown in Table 5, the highest rate of influenza vaccine uptake rate was observed among children (3–6 years = 73.3%) and health care providers (67.3%), compared to other populations [31, 46]. The lowest rate of influenza vaccine uptake was among children (6–12 month = 7.7%) and the general public (10.3%) [46, 49]. In terms of COVID-19 vaccination, the highest rate of COVID-19 vaccine uptake rate was also observed among healthcare workers (45.4%), while the lowest was among children (17.9%) [70, 71]. The highest rate of COVID-19 vaccine intent was reported among university students (71.6%) [72]. COVID-19 vaccine hesitancy was highly reported among the general public (37.7–40.9%), patients with cancer (30.6%), and refugees (25.1–50%) [73–75].

**Table 5:** Outcomes of the reviewed studies

Research study	Population	Main study outcome
Influenza vaccine		
(El Khoury & Salameh, 2015) [44]	General public: Elders; Disease-specific group	Vaccine uptake rate: 27.6%
(Taleb et al., 2018) [45]	HCWs	Vaccine uptake rate: 50.4%
(Kmeid et al., 2019) [46]	Children	6–12 months: 7.7% 1–3 year: 27.8% 3–6 year: 73.3% 6–15 year: 52.9%
(Tassi, 2020) [47]	Elders (Elder adults)	Vaccine intent: 30.4%
(Moussa, 2020) [48]	School Students	Vaccine uptake rate: 48.2%
(Choucair et al., 2021) [49]	Mix (HCWs)	Vaccine uptake rate: 10-35.6%
(Alame et al., 2021) [50]	HCW	Vaccine uptake rate: 40.4%
(Zakhour et al., 2021) [51]	School Students	Vaccine uptake rate: 29.4%
(Dalal Youssef, Berry, et al., 2022) [31]	HCWs	Vaccine acceptance: 58% Vaccine uptake rate: 67.33%
**COVID-19 vaccine**		
(Abu-Farha et al., 2021) [52]*	General Public (HCWs)	Vaccine Hesitancy: 33% Willingness 25%
(Salibi et al., 2021) [61]	Older Refugees	No intention: 28.8%
(C. Kasrine Al Halabi et al., 2021) [30]	General Public: Disease-specific group	Vaccine refusal: 40.9% Vaccine Hesitancy: 37.7%
(Sakr et al., 2021) [54]	General Public: Students; occupational groups	Vaccine Acceptance: 30.9%
(Hamdan et al., 2021) [58]	University Mixed Students	Vaccine Hesitancy: 10% Vaccine Resistance: 3%
(Nasr et al., 2021) [32]	Dentists	Vaccine Acceptance: 86%
(E. A. Qunaibi et al., 2021) [59]*	General Public: Disease-specific group	Vaccine Acceptance:26.8%
(E. Qunaibi et al., 2021) [60]*	HCW	Vaccine Acceptance:12%
(Elissar Moujaess et al., 2021) [62]	Patients With Cancer	Vaccine Hesitancy: 30.6% Vaccine Resistance: 14.4%
(Zeitoun et al., 2022) [63]	Refugees	Vaccine intention: 24.4% Vaccine Hesitancy: 25.1% Vaccine Resistance: 50.5%
(Ghaddar et al., 2022) [65]	General Public	Vaccine Hesitancy: 33.3% Vaccine unwillingness: 14.7%
(P. Hanna, A. Issa, Z. Noujeim, M. Hleyhel, & N. Saleh, 2022) [67]	General Public (Pregnant; Disease-specific group	Vaccine Acceptance: 63.4% Vaccine Registration: 57%
(Jabbour et al., 2022) [68]	University Students	Vaccine intention: 71.6%
(Karam et al., 2022) [69]	General Public: Disease-specific group	Ready to pay: 78.1%
(Khatatbeh et al., 2022) [70]*	Parents / Children	Vaccine uptake rate: 17.9%
(Dalal Youssef, Berry, et al., 2022) [31]	HCWs	Vaccine uptake rate: 45.4% Vaccine intention: 58%

Health care workers (HCW); Lebanon included as a sub-setting (*)

### Determinants of influenza and COVID-19 vaccine intent or uptake

**Fig. 2:**
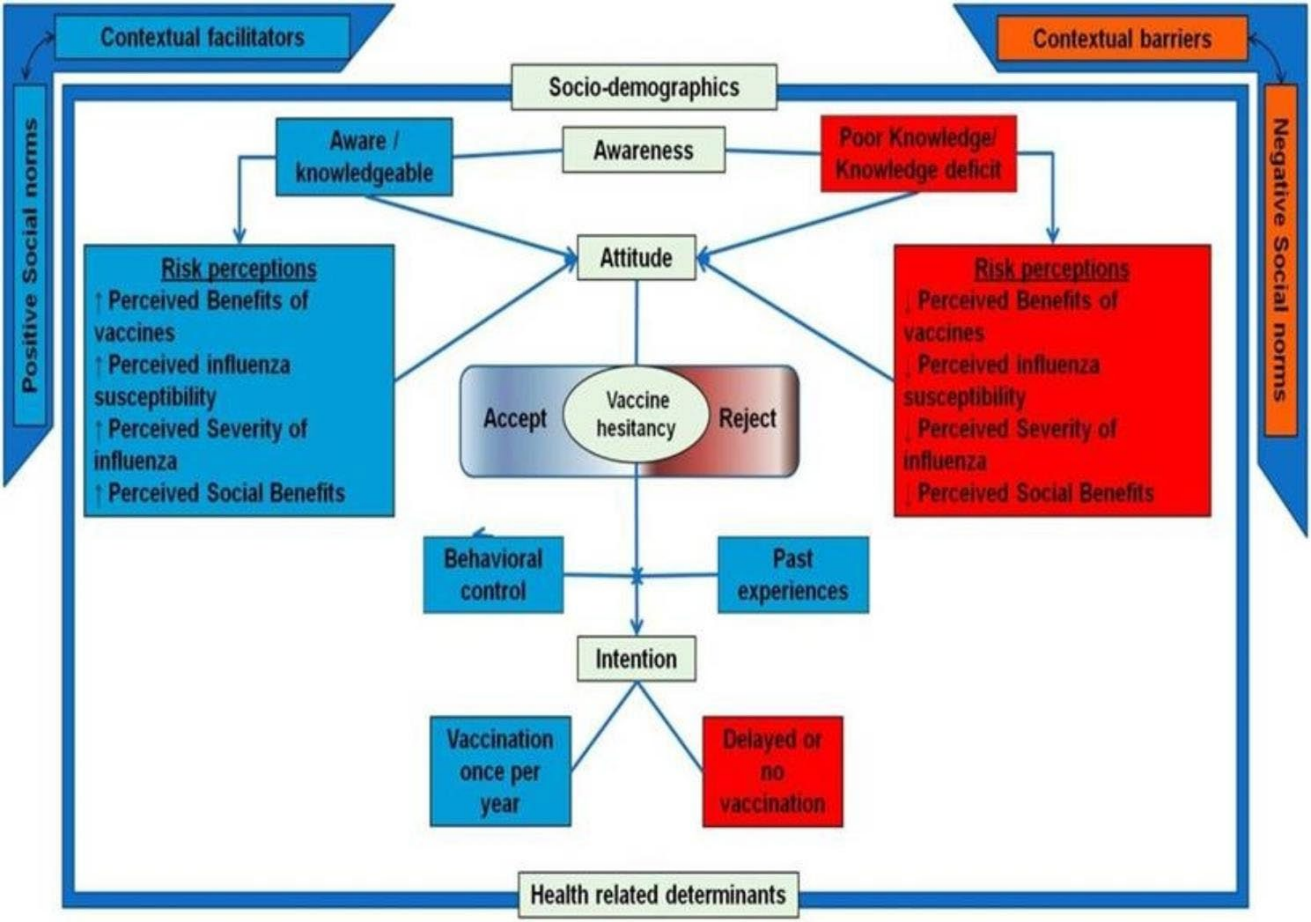
Conceptual model of influenza/COVID-19 vaccine intent and uptake in Lebanon

Determinants of influenza and COVID-19 vaccine intake or uptake in Lebanon were reported by 22 studies, whereas 9 studies did not report any determinant. Variables that were statistically significant (at the bivariate and multivariate levels of analyses) were extracted and tabulated in Table 6. Following the constructs of the TPB, these determinants were indexed under environmental constraints, norms, socio-demographics, health related factors, attitudes, personal agency, knowledge, habits and salience of behavior, and vaccine hesitancy (Table 7). All these determinants were conceptually modeled in Fig. 2.

**Table 6:** Determinants of higher influenza and COVID-19 vaccine intent or uptake rates

Influenza vaccination	
Research study	Determinants
(El Khoury & Salameh, 2015) [44]	Older age^R^; Higher educational level^R^; Having a health insurance; Good financial situation; Cigarette smoking; Alcohol drinking ^R^; Physically active ^R^; Frequent visits to a physician ^R^; Has respiratory disease ^R^; Good knowledge; Positive attitude; Sources of information (pharmacists/physcians)
(Kmeid et al., 2019) [46]	Locals (Lebanese) higher than refugees (Syrians); Child’s age(3–6 years) ^R^; Father’s age(> 31 years); Mother’s age(> 31 years); Parents with higher levels of education; Mother’s profession (private sector); Having a health insurance; Place of medical consultation / vaccination (Pediatrician clinic); Regular visit to a pediatrician; Vaccine required by school/day-care; Vaccine cost
(Tassi, 2020) [47]	Good knowledge about influenza; Believed that influenza vaccine is important for elderly adults; Believed that it should be taken yearly; Believed that it prevents serious complications; Believed that life immunity is not enough; Believed in vaccine effectiveness; Believed that influenza is a mild illness; Believed that vaccine is necessary; No fear of vaccine side effect or needles / injection; Did not adopt an alternative protective measure; Believed that the vaccine is affordable; Exposed to recommendations from doctor and patients
(Taleb et al., 2018) [45]	Younger age^R^; Convenient time; Lower perceived risks associated with vaccines’ side effects; Perceived vaccine benefits; Affordability of the vaccine; Good knowledge.
(Alame et al., 2021) [50]	Having a motive to protect themselves, community and family; Believed in boosting immunity; Believed that vaccines decrease the severity of the infection and the risk of complications; Feeling at high risk of infection; Believe in virus evolution; Did not have any vaccine efficacy concerns; Did not fear vaccine side effects; Did not belief the natural immunity is enough; Believed that the vaccine is affordable; Exposure to sources of vaccine recommendations; Good knowledge; Being a nurse; Past vaccination.
(Moussa, 2020) [48]	Good knowledge; Perceived that vaccines are safe and prevent infection; Acknowledged that the vaccine should be taken before the influenza season; Believed that vaccines are required for children, medical staff, patients with chronic diseases, and elderly; Being worried about influenza; Believed that the vaccine is a protective strategy against influenza infection; Being advised by families and friends to take the vaccine; Exposed to doctor’s recommendation; Believed that influenza is serious
(Zakhour et al., 2021) [51]	Younger age; Higher school level; Paternal employment; Higher income; Exposed to awareness campaigns by SMS; Exposed to doctors’ recommendations; Perceived vaccine safety and efficacy; No vaccine misconceptions; Good knowledge; Had vaccine trust; No hesitancy; No concern about side effects.
(Choucair et al., 2021) [49]	Being a healthcare worker; Older age; Being married; Having children^R^; Self-rated good knowledge about influenza^R^; Exposed to vaccine recommendations by physician; Higher perceived risk of influenza^R^
**COVID-19 vaccination**	
Research study	Determinants of study outcomes
(Dalal Youssef, Berry, et al., 2022) [31]	Female gender ^R^; Health care worker working in the frontline ^R^; Previous influenza vaccination ^R^; Living in urban areas^R^; Previously diagnosed with COVID-19^R^; Disagreed on the idea of novel vaccine^R^; Believed in vaccine safety^R^; Believed in the reliability of manufacturer^R^; Vaccine accessibility and availability^R^; Exposure to reliable and adequate information about the vaccine^R^; Exposure to recommendations by health authorities, health facilities and a family member^R^; Good perceived COVID-19 vaccine benefits: protects them and their close contacts^R^
(Abu-Farha et al., 2021) [52]	Nationality(Highest in Saudi Arabia); No issues with vaccine cost; Preferred Pfizer-BioNTec; Exposed to source of information (governmental), Holding a medical related degree^R^; Higher income^R^; Being married^R^; Country of residence(Saudi Arabia)^R^; Having received influenza vaccine previously ^R^; Low fear score ^R^; feeling at risk of getting infected with COVID-19^R^; Female gender ^R^.
(Salibi et al., 2021) [61]	No issues with the novelty of the vaccine; No need to wait to know more about it; Preference to maintain public health precautions; Belief that vaccines are essential; Not worried about vaccine side effects; Trust in the health care system; Residing in a formal settlement in a host country ^R^; Good perceptions of vaccine safety ^R^ and effectiveness ^R^.
(C. Kasrine Al Halabi et al., 2021) [30]	Male gender ^R^; Single marital status ^R^; Lower hesitancy scores.
(Hamdan et al., 2021) [58]	Adherence to universal precautions; Previous influenza vaccination; Did not believe in conspiracy beliefs (Commercial profiteering, manipulation by a higher power, the vaccine taking away personal freedom and governmental control); Good knowledge (transmissibility) ^R^; Perceived vaccine safety ^R^; Pro-vaccine descriptive norms ^R^
(Elissar Moujaess et al., 2021) [62]	If the vaccine follows international recommendations; If the vaccine did not interfere with their treatment; Desire to know more about the consequences of the vaccine in other patients with cancer; Considered themselves more vulnerable to COVID-19
(Nasr et al., 2021) [32]	Desire to protect their themselves and families; Wanting the pandemic to end quickly; Desire to return to their normal social life; Not concerned about the possible long-term side effects of COVID-19 vaccines or the rapidity of vaccines’ development; Believed in the effectiveness of the vaccine against new viral variants; Good knowledge ^R^; Previous influenza vaccine uptake during the COVID-19 ^R^; Visiting the medical doctor ^R^
(Ghaddar et al., 2022) [65]	Young age group; lower level of education; Being employed; Trust in news from the WHO, MoPH and radio/TV with statistically significant differences; Male gender^R^; Having trust in Facebook^R^; Not affected by fake news ^R^; Do not belief in the man-made theory or the business control theory ^R^; Trust in news from MoPH^R^
(Karam et al., 2022) [69]	Higher level of education; Higher salary; Perceived severity of COVID-19
(Philippe Hanna et al., 2022) [76]	Believed in the need to protect themselves and family members from getting infected by COVID-19; Driven by the need to end the pandemic quickly, to return to normal life with family and friends; No Barriers: no concern about the potential long-term serious side effects; Not concerned about the short time allocated to vaccines’ clinical trials; Believed in the new technologies (mRNA) used in the vaccine production; No fear of short-term side effects (allergic reactions); Need to observe positive vaccines effects in people they know; Received more information about COVID-19 vaccines; Vaccine brand; Good knowledge index ^R^; Hypertension as a co morbidity ^R^; Higher fear scale ^R^; Past influenza vaccination ^R^; Living in rural areas ^R^; Reported food allergy ^R^
(Jabbour et al., 2022) [68]	Reading about the safety and effectiveness of COVID-19 vaccine; Perceived vaccines as safe or effective; Positive social media impact; Healthcare workers and those who believed that most people sharing posts try to be honest^R^; Positively associated with the use of Facebook^R^; Social media trust^R^
(Sakr et al., 2021) [54]	Education ^R^; Perceived benefits of influenza vaccines to self or community ^R^
(Zeitoun et al., 2022) [63]	Recommended by government/MOH/CDC; Perceived severity of viral influenzas; Good knowledge; Exposure to sources of information; Having an access to vaccines; Pace of vaccine development; Influenced by social media; Influenced by research; Social influences; Female gender; Place of birth/immigration; Religious affiliation / cultural; Using alternative protective measures; Vaccine is the best preventive measure; Low hesitancy scores; Believed that vaccine do not changes in DNA
(Dalal Youssef, Abou-Abbas, et al., 2022) [77]	Perceived benefits of influenza vaccines ^R^; Recommended by government; Perceived severity of viral influenzas ^R^; Knowledge ^R^; Source of information; Access to vaccines ^R^; Clinical trials ^R^ (no sufficient studies); Recommended by HCWs; Perceived susceptibility to vaccine side effects; Information seeking ^R^; Gender ^R^; Occupation ^R^; Urban vs. rural ^R^; Previous history of influenza; FDA approval ^R^; Recommended by HCWs ^R^; Motivated by patient protection; Physical activity

^R^: Statistically significant at the level of regression analyses

**Table 7:** Determinants of influenza and COVID-19 vaccine intent or uptake in Lebanon indexed under the model constructs

Influenza vaccine studies	Environmental constraints	Perceived norms	Salience of behavior	Sociodemographics / health related factors	Knowledge and skills	Personal agency	Attitudes and risk perceptions	Vaccine hesitancy
(El Khoury & Salameh, 2015) [44]	X	X		X	X		X	
(Kmeid et al., 2019) [46]	X	X		X				
(Tassi, 2020) [47]	X	X			X	X	X	
(Taleb et al., 2018) [45]	X			X	X		X	
(Alame et al., 2021) [50]	X	X	X	X	X		X	
(Moussa, 2020) [48]		X			X	X	X	
(Zakhour et al., 2021) [51]	X	X		X	X		X	X
(Choucair et al., 2021) [49]		X	X	X	X		X	
**COVID-19 vaccine studies**								
(Dalal Youssef, Berry, et al., 2022) [31]	X	X	X	X	X		X	X
(Abu-Farha et al., 2021) [52]	X		X	X	X			X
(Salibi et al., 2021) [61]	X				X	X	X	X
(C. Kasrine Al Halabi et al., 2021) [30]				X				X
(Hamdan et al., 2021) [58]		X	X		X	X	X	X
(Elissar Moujaess et al., 2021) [62]					X			X
(Nasr et al., 2021) [32]		X			X			X
(Ghaddar et al., 2022) [65]				X	X	X		X
(Karam et al., 2022) [69]	X			X			X	
(Philippe Hanna et al., 2022) [76]	X	X		X	X	X	X	
(Jabbour et al., 2022) [68]		X		X			X	
(Sakr et al., 2021) [54]				x			x	
(Zeitoun et al., 2022) [63]	x	x		x	x		x	x
(Dalal Youssef, Abou-Abbas, et al., 2022) [77]	x	x		x	x		x	

The most frequently reported determinants of influenza and COVID-19 vaccine intent or uptake were the “Perceived benefits of vaccines to self or community” (n = 7 studies) and the “Perceived susceptibility to vaccine side effects” (n = 6 studies), followed by the “Source of information about vaccines” (n = 5 studies), the “Perceived severity of viral influenzas” and the “Knowledge on vaccine benefits, indications to vulnerable groups” (n = 4 studies each). Other determinants -stratified by the vaccine type- are presented in Table 8.

**Table 8:** Determinants of vaccine intent or uptake stratified by the vaccine type

	Influenza vaccine (n)	COVID-19 vaccine (n)
Contextual determinants		
Access to vaccines/ unavailability	1	1
Mandates/ policies (presence or the absence of them)	2	0
Need to travel	0	1
Social norms and Cues to action		
Social influences (community and school)	0	1
Influenced by social media / videos / platforms / websites (trusting them)	0	2
Recommended by physicians, pharmacists, or other HCWs	1	0
Recommended by governmental services or international organizations (MOH/CDC)	1	1
Influenced by research and evidence based findings / studies	0	1
Influenced by mail reminders	0	3
Motivated by patient protection,	1	1
Risk perceptions and Attitudes		
Perceived susceptibility to vaccine side effects	3	3
Perceived susceptibility to disease	2	0
Perceived benefits of vaccines to self or community	3	4
Opposes taking medications/vaccines	2	1
Low risk perception of disease	1	1
Self perception of health (health motivation)	2	0
Perceived severity of viral influenzas (not serious to require vaccination)	1	3
Waiting for others to get vaccinated	0	1
Health related determinants	1	1
Knowledge and awareness		
Knowledge on vaccine benefits, indications to vulnerable groups, frequency	2	2
Source of information / not enough / trusting the source	2	3
Knowledge on the annual shot / duration / frequency	1	0
Knowledge on the availability of vaccines / types of vaccines	1	0
Information seeking / reading about the topic	0	1
Socio-demographics		
Gender	0	1
Age	2	1
Place of birth; immigration status	0	2
Marital status	1	0
Level of education or place of education	0	1
Religious /cultural affiliation	0	1
Work area (Medical center, nursing home, university), workload	1	0
Race; ethnicity; nationality; languages	0	1
Financial status; Socio Economic Status; Free vaccine	0	1
Occupation (rank, position, healthcare provider vs. not); exposure to COVID-19 cases	2	1
Having children	2	0
Past experiences with diseases and vaccines		
Not using alternative protections to vaccines (washing hands, avoid crowds, etc.) - vaccine is best in prevention	0	2
Vaccine hesitancy	0	1
Vaccine related factors		
Clinical trials (no sufficient studies), rapid vaccine development	0	3
Vaccine administration / site of injection	0	1
Vaccine manufacturers / Government	1	0
Type of vaccine: mRNA, attenuated	0	1
Barrier		
No time / busy	1	0
Fear of needles / pain	1	0
Cost	1	0
Misconceptions		
Conspiracy / ulterior motives / human made virus	0	1
Herbal drinks are better	0	1
Vaccine changes our DNA	0	1

n = number of studies

The conceptual model in Fig. 2 illustrates the relationship between various determinants of influenza and COVID-19 vaccine intent or uptake. At the interpersonal level, contextual determinants include the access to vaccines, national vaccine policies/ campaigns, and the health care systems that deliver them. At the same level, a set of social norms and influences play a role in the individual’s decision making to get vaccinated (influence of the community, social media, recommendations of physicians, pharmacists, or others) and other factors showed in Table 8. These factors contribute to raising awareness about influenza and COVID-19 seriousness, about vaccine benefits, and about the sources of vaccines. Socio-demographic and health related determinants are universal variables that should be analyzed when investigating influenza or COVID-19 vaccine intent or uptake. For instance, being an elderly individual or having pre-existing medical conditions are expected to be more concerned about influenza/COVID-19, so they are more likely to seek vaccination. At the intrapersonal level, awareness is the individuals’ familiarity with the disease and vaccines on different aspects (vaccine benefits/indications, frequency of taking it and vaccine providers). Knowledge affects perceptions, i.e. the perceived severity/susceptibility of influenza/COVID-19 and the perceived benefits of vaccines. At this stage, individuals are expected to engage in a transient decision making stage during which they can accept, hesitate or resist influenza /COVID-19 vaccines. During this stage, the individuals’ past experiences and their level of control over their actions (vaccine affordability and accessibility) influence their decisions and might facilitate/delay the period between their intention to get vaccinated and the vaccine uptake.

## Discussion

Determinants of influenza and COVID-19 vaccine intent or uptake are discussed at the interpersonal and intrapersonal levels, and then compared to findings from neighboring countries. Implications to practice and research are stated throughout this section.

### Determinants at the interpersonal level

Contextual factors, such as vaccine availability, accessibility, and affordability, are usually specific to a setting or population that might play either a facilitating or a hindering role in the uptake of influenza or COVID-19 vaccines [29]. For instance, some residents in Lebanon complained of unavailable certain types of COVID-19 vaccines, which delayed their COVID-19 vaccine uptake [77]. Even in high income MENA countries like the United Arab Emirates, vaccine unavailability issues were reported in some regions [78]. Low influenza or COVID-19 vaccine uptake might be due to a limited access to the healthcare systems [29, 61]. Previous studies from Saudi Arabia and Turkey showed that individuals who have access to a primary health care center are more likely to interact with healthcare workers and get advised on influenza and COVID-19 vaccine uptake [79, 80]. Lebanon has primary care centers distributed across its eight provinces, but transportation might be a constraint to some people [81]. In terms of vaccine affordability, influenza vaccine in Lebanon costs USD18, while COVID-19 vaccines are currently provided for free [50, 82]. Waiving off influenza vaccine costs was associated with higher vaccine uptake rates in Jordan where healthcare workers benefited from 80 to 90% discounts on influenza vaccine costs [83]. Therefore, a convenient access to clinics, affordable vaccines, and empowerment positively contribute to a higher vaccine uptake [29, 84].

Social norms that are pro vaccines were associated with higher vaccine uptake rates [29, 58]. For instance, being a requirement by some Lebanese schools made it a norm for parents to vaccinate their children against influenza every year [46]. On the other hand, healthcare workers from Turkey stated that social influences affected their decision to get vaccinated against influenza [85]. Healthcare workers are always reminded about the importance of getting vaccinated as a protective strategy for themselves, their community and families [31, 50]. A salient vaccination behavior is the one that is widely observed within clustered communities or confined within a period of time [52]. For instance, in terms of COVID-19 vaccines, one of the negative prevailing norms in Jordan and Saudi Arabia was “waiting for others to get vaccinated” [86, 87]. Furthermore, vaccination drives are launched prior the influenza season every year, and it is not uncommon for many individuals to seek vaccination in groups [49, 50]. Mass scale vaccination campaigns have been associated with higher vaccine uptake rates in Saudi Arabia [88]. This will probably encourage unvaccinated individuals to reconsider their decisions [31, 49].

The relationship between socio-demographics and vaccine uptake was inconclusive across many studies, despite being important when surveying homogeneous populations [29]. This relationship is probably confounded by certain contextual factors or mediated by certain psychological factors such as risk perceptions [31, 50]. For example, older Lebanese adults regularly sought influenza vaccination due to their heightened risk perception of contracting influenza/COVID-19 and interaction with health care practitioners during which they get advised on vaccines [44]. In many Arab populations, the intention to get vaccinated against COVID-19 was higher among the younger Jordanian, Palestinian and Syrian adults (18–35 years old) compared to older age groups, whereas the rate of COVID-19 vaccine intention was higher among Israeli older adults [89, 90]. The individual’s health condition and lifestyle characteristics such as smoking, alcoholism, and physical inactivity were associated with influenza vaccine uptake [44, 51]. For instance, a study from Kuwait stated that non-smokers reported lower perceived risk of contracting COVID-19 [91]. Some researchers noted that patients had more desire to get vaccinated compared to students and health practitioners probably due to their pre-existing medical conditions [92], or since they regularly interact with their healthcare providers which makes them routinely exposed to medical advise [29]. Even within the same academic institute, students from different faculties differed in their COVID-19 vaccine intention rates [93].

### Determinants at the intrapersonal level

Knowledge about influenza/COVID-19 infections (etiology, method of transmission, signs/symptoms) and its vaccines (indications, frequency of taking it and providers) is a key determinant of vaccine uptake [29, 44, 47]. Besides the theoretical knowledge, individuals also rely on past experience with getting infected with influenza/COVID-19 and vaccines before making their decision to get vaccinated [31, 50]. In the absence of this knowledge or past experience, individuals are not expected to engage in the decision making process to take the vaccine. A study from Pakistan showed that postpartum women who experienced COVID-19 were more likely to accept the COVID-19 vaccine than women with no prior experiences [94]. Students from UAE showed that those who reported being previously infected by COVID-19 reported higher intent rates to receive the vaccine compared to their counter group [95]. Raising awareness triggers a transient decision making process [31, 44, 50, 51]. One study showed that 56.8% of the Lebanese public were aware of influenza vaccine benefits, yet they did not regularly seek influenza vaccination [44].

The source of information and the level of trust in these sources also play a role in vaccine uptake [31, 51, 52, 61, 65]. For instance, the Lebanese who sought information about influenza vaccines from health practitioners and family members were more likely to get vaccinated [44, 92], while those who sought information from the social media were reluctant [44]. Parents of children who received adequate information about COVID-19 vaccines reported higher intent rates to vaccinate their children in five Arab countries in the Middle East [96]. Knowledge about influenza/COVID-19 and their vaccines are expected to enhance the individual’s control over their vaccine behavior that is their ability to overcome any barrier to vaccination [29]. Past experience and knowledge both play a facilitating role in overcoming these barriers [29, 31, 51]. Last but not least, behavioral control becomes more stringent when individuals interact with healthcare practitioners and follow their recommendations [29].

Cognitive risk perception dwells on how individuals evaluate the risk of contracting influenza, while emotional risk perception is how much individuals worry about contracting influenza or receiving its vaccine [50]. A perceived relative risk entails how individuals compare themselves to others in relation to these risks [29, 97]. High risk perception related to contracting influenza/COVID-19 was associated with high vaccine uptake in many MENA countries [98, 99]. Confronting some individuals -such as the health care workers - with the fact that they might transmit influenza or COVID-19 to their patients have been often used as an ethical argument to get them vaccinated [100]. In Saudi Arabia, the most frequently reported motivation to get vaccinated among healthcare workers were ‘self and ‘patient protection’ [101].

When attitudes are not in favor of vaccines, it means that vaccines are not important enough to actively overcome any barrier [29]. Some individuals might have negative pre-existing attitudes towards influenza vaccines, yet base their decisions on “utility maximization” i.e. if the vaccine was conveniently provided to them [102]. For instance, Saudi Arabian healthcare workers reported vaccine convenience as an important factor in vaccine decision-making [102].

Healthcare workers usually report better attitude towards influenza vaccines compared to the general public [50]. Negative attitude towards COVID-19 vaccines is sometimes based on the unforeseen / undiscovered problems of vaccines or unresolved suspicions in pharmaceutical companies / health authorities [58]. However, the severity of COVID-19 pandemic -compared to seasonal influenza- played a detrimental role in enhancing the individuals attitude towards COVID-19 vaccines [69]. Therefore, informing individuals about both the personal and societal benefits of vaccines (herd immunity) can play a role in enhancing the individual’s attitude towards vaccines [29].

Vaccine hesitancy or reluctance overlaps with intention and it might delay or prevent vaccination [103]. It is a transient state of internal cognitive / emotional conflict that hinders the translation of intention to an actual behavior [103]. If an individual is not confident in the vaccines’ benefits or the system that provides them, they are more likely to become hesitant and delay vaccination [104]. In Tunisia, patients with cancer reported less hesitancy towards vaccines since they considered vaccines a gateway towards ending the pandemic [105]. However, if some individuals get vaccinated it doesn’t imply that their hesitancy is resolved [103]. Therefore, the intention to get vaccinated or even vaccine uptake should not be accounted as an indicator of vaccine acceptance. This is important as vaccination is recommended every year, and vaccine-hesitant individuals might refrain from seeking vaccination in the upcoming year, even if they get vaccinated at present.

### Strengths and limitations

This scoping review study has to be seen in light of few strengths and limitations. Evaluating the current body of research on influenza and COVID-19 vaccines in Lebanon serves as a guide to future researchers by identifying knowledge gaps, research priorities, under-researched settings and populations. This review probably presents the first conceptual model on influenza and COVID-19 vaccine uptake within the Lebanese conext. This review also exposed the commonly used research measures and theoreticl models followed by the Lebanese researchers who investigated influenza and COVID-19 vaccines. In addition, this review presented a list of journals that researchers can consider when planning to publish their research findings.

Few limitations can be reported in this scoping review. In terms of space, the search strategy was limited to one country which might limit the generalizability of its findings elsewhere. However, the Lebanese population share alot of common features with populations residing in its neighboring countires, such as Syria and Jordan. In terms of time, the rate of publications peaked in 2022 and it is expected to show a steady increase in the coming years due to the emergence of COVID-19. Many of the captured studies in this review investigated COVID-19 vaccine intent rates compared to studies on COVID-19 vaccine uptake rates. It is expected that the latter studies will continue to present more updated information on the COVID-19 vaccine status in Lebanon. This can be addressed in future review studies and the proposed model can be adjusted accoridngly. The protocol for this scoping review study was revised by a PhD thesis committe. It was not registered at the international database of Prospectively Registered Systematic Reviews (PROSPERO) since they do not accept the registration of scoping review studies.

## Conclusions and research propositions

Research on influenza/COVID-19 vaccine uptake in Lebanon is still in its infancy. This review conceptualized vaccine uptake within the Lebanese context and presented its multifaceted determinants. Few attempts were made by the Lebanese researchers to target vulnerable non-health occupational groups, as per the National Institute for Occupational Safety and Health, such as public administration, educational services, transportation, accommodation and food services [106]. Therefore, there is a need to evaluate the influenza and COVID-19 vaccine uptake within vulnerable non-health occupational groups. Fundamental causes of vaccine hesitancy were not investigated in Lebanon. This needs macro-level studies to compare the determinants of vaccine uptake across populations that vary by their socioeconomic status, nativity, area of residence and health care privileges. The multiple crises that Lebanon has witnessed lately have deteriorated the socio-economic status in the country. This threatened a sustainable service provision (limited vaccine supplies) and made some Lebanese incapable of purchasing vaccines [29]. Evaluating the effectiveness of vaccination programs also requires a multi-country study to compare the Lebanese vaccination programs to international ones.

## Data Availability

The datasets used and/or analysed during the current study are available from the corresponding author on reasonable request.

## List of Abbreviations

COVID-19: Corona Virus Infectious Disease
MoPH: Ministry of Public Health
MENA: Middle Eastern and North African
WHO: World Health Organization
CDC: Centers for Disease Control and Prevention
URLs: Uniform Resource Locators
PRISMA: Preferred Reporting Items for Systematic Reviews and Meta-Analyses
